# Composition and diversity of rhizosphere fungal community in *Coptis chinensis* Franch. continuous cropping fields

**DOI:** 10.1371/journal.pone.0193811

**Published:** 2018-03-14

**Authors:** Xuhong Song, Yuan Pan, Longyun Li, Xiaoli Wu, Yu Wang

**Affiliations:** 1 Chongqing Academy of Chinese Materia Medica, Chongqing, China; 2 Chongqing Engineering Research Center for Fine Variety Breeding Techniques of Chinese Materia Medica, Chongqing, China; 3 Chongqing Sub-center of National Resource, Center for Chinese Materia Medica, China Academy of Chinese Medical Science, Chongqing, China; USDA Forest Service, UNITED STATES

## Abstract

In this study, effects of continuous cropping on soil properties, enzyme activities, and relative abundance, community composition and diversity of fungal taxa were investigated. Rhizosphere soil from field continuously cropped for one-year, three-year and five-year by *Coptis chinensis* Franch. was collected and analyzed. Illumina high-throughput sequencing analysis showed that continuous cropping of *C*. *chinensis* resulted in a significant and continuous decline in the richness and diversity of soil fungal population. Ascomycota, Zygomycota, Basidiomycota, and Glomeromycota were the dominant phyla of fungi detected in rhizosphere soil. Fungal genera such as *Phoma*, *Volutella*, *Pachycudonia*, *Heterodermia*, *Gibberella*, *Cladosporium*, *Trichocladium*, and *Sporothri*x, were more dominant in continuously cropped samples for three-year and five-year compared to that for one-year. By contrast, genera, such as *Zygosaccharomyces*, *Pseudotaeniolina*, *Hydnum*, *Umbelopsis*, *Humicola*, *Crustoderma*, *Psilocybe*, *Coralloidiomyces*, *Mortierella*, *Polyporus*, *Pyrenula*, and *Monographella* showed higher relative abundance in one-year samples than that in three-year and five-year samples. Cluster analysis of the fungal communities from three samples of rhizosphere soil from *C*. *chinensis* field revealed that the fungal community composition, diversity, and structure were significantly affected by the continuous cropping. Continuous cropping of *C*. *chinensis* also led to significant declines in soil pH, urease, and catalase activities. Redundancy analysis showed that the soil pH had the most significant effect on soil fungal population under continuous cropping of *C*. *chinensis*.

## Introduction

*Coptis chinensis* Franch. is one of the most important traditional medicinal plants in the family Ranunculaceae. The root of *C*. *chinensis* is regularly used for medicinal purposes, and has been prescribed alone or in combination with other traditional herbs for treating diabetes, dysentery, jaundice, acute febrile and suppurative infections, seasonal febrile diseases, sore throat, reducing fever, and preventing diarrhea [1]. Previous phytochemical studies on *C*. *chinensis*, detected more than 30 alkaloids [2]. Among these alkaloids, berberine, epiberberine, palmatine, coptisine, and jatrorrhizine, which are isoquinoline alkaloids that are predominantly bioactive, have been confirmed as the main constituents, of the total alkaloid fraction, comprising more than 80%. Isoquinoline alkaloids obtained from herbal extracts of *C*. *chinensis* were found to display multiple biological activities, such as broad-spectrum antimicrobial [3], anti-inflammation [4–7], anti-cancer [8], anti-diabetes [9–12], attenuation depressive-like behaviors [13], Anti-adipogenesis [14], enhancement of osteogenic differentiation [15], reduction of oxidative stress [16] and anti-phototoxicity [17] effects.

In China, *C*. *chinensis* is only distributed in Guizhou Province, Sichuan Province, Chongqing City, Shanxi Province, and Hunan Province. Currently, the most well-known crop area is Shizhu Tujia Autonomous County, which lies in Chongqing City. At present, the production of medicine from *C*. *chinensis* mainly depends on the field cultivation of the species. Given the increased demand for *C*. *chinensis* for medicinal purposes, continuous cropping of the herb has become increasingly common. Long-term continuous cropping of Chinese medicinal herbs often leads to a decrease in plant growth, serious root rot disease, and considerable yield loss [18]. Continuous cropping of *C*. *chinensis* resulted 70% to 80% reduction in yield. At the same time, root rot disease has become the major threat to the production of *C*. *chinensis* [19].

Soil microbial and biochemical properties are important soil health indicators because they are involved in soil organic matter decomposition and nutrient availability and cycling [20,21]. Soil enzyme activities and microbial biomass pools have also been proposed as integrative indicators of soil quality [21,22]. More recently, an increasing number of studies have speculated that continuous cropping or consecutive monoculture resulted in imbalances in soil microbial community diversity and structure [23–25]. Moreover, fungal pathogen populations increase rapidly in soil under continuous cropping [24,26–29].

Several molecular analytical technologies are used to analyze the diversity of soil microbes in continuous cropping systems, such as 454 pyrosequencing analysis [18,27], denaturing gradient gel electrophoresis [30,31], terminal restriction fragment length polymorphism [28], and fluorescence in situ hybridization [32]. However, these molecular technologies cannot overcome the shortage of species and generally fail to provide a sufficient amount of data for analysis within a short time. Recently, the high-throughput sequencing approach has been frequently used to detect the diversity of rhizosphere soil microbes as well as their relative abundance and evolution [33–37].

Previous studies have shown that the average incidence of root rot in *C*. *chinensis* fields was 11.4%, 38.7%, and 21.5% for one-year three-year and five-year continuous cropping of *C*. *chinensis*, respectively. The present study was conducted using the high-throughput sequencing technique (Illumina HiSeq 2500 PE250 Platform) to evaluate the rhizosphere soil fungal abundance and community composition changes with increasing years of cropping. The effect of soil properties on fungal community structure was investigated. Furthermore, the factors contributing to the incidence of root rot in one-year, three-year and five-year continuously cropped *C*. *chinensis* were explored.

## Materials and methods

### Soil sampling

The soil samples for the present investigation were collected from the cropping fields of *C*. *chinensis* present in Shazi Town, Shizhu Tujia Autonomous County, Chongqing City, China (108°26′ E, 29°59′ N). The altitude, annual rainfall and mean annual temperature of the study area are 1,667 m, 1,200 mm, and 16 °C, respectively. The soil type of the local area is sandy loam soil. The rhizosphere soil samples collected from the fields, continuously cropped with *C*. *chinensis* for one-year, three-year and five-year have been denoted as RMS1, RMS3 and RMS5, respectively. All the three treatments were arranged in the same area and conducted under uniform field conditions and management. Each treatment had the three replicate plots selected randomly. Each plot was 20 m^2^ and contained 780 plants. These plants were kept 15 cm apart from each other. On August 5, 2015, rhizosphere soil samples were collected from 30 randomly selected plants in one plot. The roots were shaken gently to remove the loosely adhering soil. Then, the rhizosphere soil that clung to the roots and rhizomes of *C*. *chinensis* was brushed off and collected. The collected rhizosphere soil from the three replicate plots was thoroughly mixed as a composite sample. A part of each soil sample was stored in an ultralow temperature freezer at −80 °C for DNA extraction, and the remaining soil was air-dried for soil characteristic and enzyme activity analyses [27,38].

### Total DNA extraction from rhizosphere soil

The total genomic DNA from the samples was extracted from 1 g soil using the Fast DNA Spin Kit for Soil (MP Biomedicals, Santa Ana, CA, USA) according to the manufacturer’s instructions. DNA extracted for 30 times from every treated soil sample was mixed as one DNA mixed sample. Then, the three mixed DNA samples were used for the fungal internal transcribed spacer (ITS) gene amplification. DNA concentration and purity were monitored on a 1% agarose gel. Based on the concentration, the DNA was diluted to 1 ng.μL^−1^ and stored at −80 °C until use.

### Fungal ITS gene amplification and Illumina sequencing

We consulted the protocol described by Caporaso et al. [33] to analyze the diversity and composition of the fungal communities in rhizosphere soil under continuous cropping. Primers ITS5-1737F and ITS2-2043R were selected to amplify the ITS1 region of the fungal genes. DNA was amplified following a previously described protocol [39].

### Rhizosphere soil enzyme activities and physiochemical properties

The soil pH was tested with a Mettler-Toledo TE 20 (Mettler-Toledo Instruments Co., Ltd., Shanghai, China) using soil suspension with deionized distilled water (1:20 w/v). The rhizosphere soil enzyme activities were determined using an enzyme analysis kit (Suzhou Comin Biotechnology Co., Ltd., Jiangsu, China). The soil physiochemical properties were analyzed according to Bao [40] and are described briefly as follows. The soil organic matter was measured with the potassium dichromate internal heating method. The soil alkaline-hydrolyzable nitrogen was measured with the alkaline-hydrolyzable diffusion method. The available phosphorus was extracted with 0.05 mol L^−1^ HCl–0.025 mol L^−1^ (1/2 H_2_SO_4_) and determined by molybdenum and antimony analyses. The available potassium was extracted with neutral NH_4_Ac and then measured by flame photometry.

### Statistical analysis

The paired-end reads from the original DNA fragments were merged using FLASH [36] and assigned to each sample according to the unique barcodes. Sequences were analyzed using the QIIME (version 7.0) [33] software package, and in-house Perl scripts were used to analyze alpha diversity. Sequences with ≥97% similarity were assigned to the same operational taxonomic unit (OTU). We selected representative sequences for each OTU and used the RDP classifier [41] to annotate the taxonomic information for each representative sequence. The observed species richness, Shannon diversity index, ACE, and Good’s coverage index were used to calculate the alpha diversity with QIIME and were displayed with R software (version 2.15.3). Rarefaction curves were generated based on the observed species richness. The Venn diagram was used to display the common and unique OTUs among the three soil samples. Spearman rank analysis, redundancy analysis (RDA), and the generation of a heat map of the 35 most abundant fungal genera were performed using the R software.

The soil physiochemical properties and rhizosphere soil enzyme activities were analyzed by performing a one-way ANOVA as implemented with using the SPSS statistical software (version 16.0; IBM, Armonk, NY, USA).

### Sequence accession numbers

All raw sequence data are accessible in the NCBI Sequence Read Archive (SRA) database under accession number SRR5248582.

## Results

### Physicochemical characteristics and enzyme activities of rhizosphere soil

The physiochemical characteristics of soil during the continuous cropping of *C*. *chinensis* are shown in Table 1. Overall, the soil pH declined continuously as the number of planted years increased. The soil organic matter, alkaline-hydrolyzable nitrogen, available phosphorus and available potassium all increased with the increase in the number of continuous planted years.

**Table 1:** Physiochemical characteristics of rhizosphere soil under *C*. *chinensis* continuous cropping.

Sample	pH value	Soil organic matter (g.kg^-1^)	Alkaline-hydrolyzable nitrogen (mg.kg^-1^ soil)	Available phosphorus (mg.kg^-1^ soil)	Available potassium (mg.kg^-1^ soil)
**RMS1**	5.60a	11.34b	160.04c	77.55c	156.38c
**RMS3**	5.46b	12.87a	176.07b	130.54b	312.79a
**RMS5**	5.08c	12.36a	199.70a	146.09a	183.61b

Values are means ± standard error (n = 3). Means followed by the same letter for a given factor are not significantly different (*P* < 0.05; LSD test).

The soil enzyme activities of sucrase, urease, cellulase, polyphenol oxidase, and catalase were determined to measure the potential turnover rates of nitrogen and carbon (Table 2). The enzyme activities of urease and catalase showed a sustained significant reduction with the increase in the number of planting years (*P* < 0.05). The activities of sucrase, cellulase and polyphenol oxidase in RMS5 were significantly higher than those in RMS1 or RMS3 (*P* < 0.05).

**Table 2:** Enzyme activities of rhizosphere soil.

Sample	Sucrase (mg.d^-1^.g^-1^)	Urease (μg.d^-1^.g^-1^)	Cellulase (mg.d^-1^.g^-1^)	Polyphenol oxidase (mg.d^-1^.g^-1^)	Catalase (μmol.d^-1^.g^-1^)
**RMS1**	27.68b	416.56a	16.35b	44.63b	37.48a
**RMS3**	25.34b	148.47b	17.17b	42.07b	28.74b
**RMS5**	37.36a	127.31c	18.99a	53.83a	24.74c

Values are means ± standard error (*n* = 3). Means followed by the same letter for a given factor are not significantly different (*P* < 0.05; LSD test)

### Fungal species richness and alpha diversity

**Fig 1:**
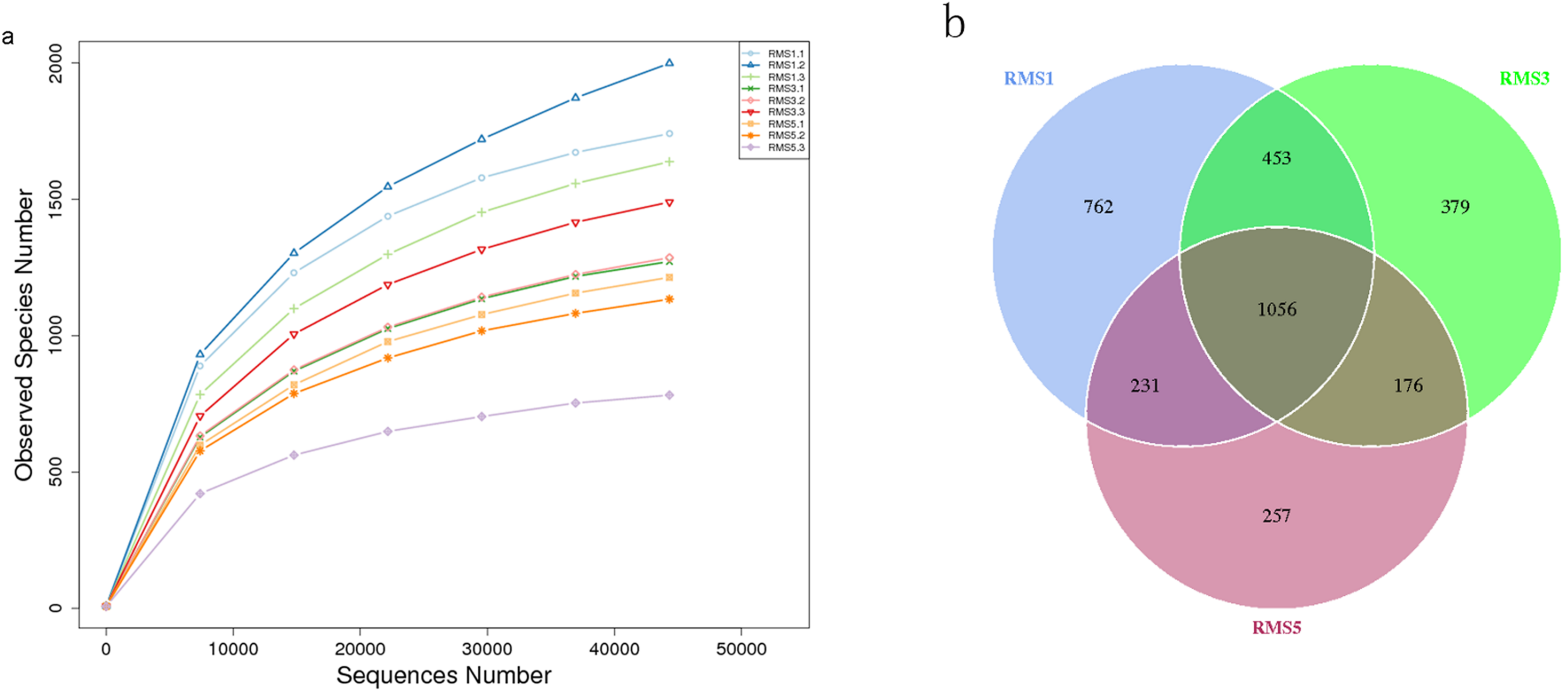
Rarefaction curves and Venn diagram. a. Rarefaction curves for three samples at an OTU threshold of 97% sequence similarity; b. Venn diagram for the three collected rhizosphere samples of soil under continuous cropping of *C*. *chinensis*.

The results of rarefaction curves can directly reflect the ordering of the volume of sequencing data and indirectly reflect the richness of species across all samples. Fig 1a shows that the curve tends to flatten out, indicating that the sequencing data volume is reasonably ordered and that more data would not result in more OTUs. More than 477,898 effective tags were obtained (i.e., 154,043 for RMS1, 160,253 for RMS3, and 163,606 for RMS5) (S1 Table). The tags effective for all the samples were clustered at 97% sequence consistency (i.e., identity) to analyze the species composition and diversity of the samples. The sequence cluster was considered the OTUs. A total of 2,502, 2,064, and 1,720 OTUs were obtained from RMS1, RMS3, and RMS5, respectively (Fig 1b).

The alpha diversity of the three samples is presented in Table 3. The observed species richness, Shannon diversity index and ACE declined significantly with the increase in the number of continuous cropping years (*P* < 0.05). This result indicated that a decrease in fungal species richness and diversity with increasing planting years.

**Table 3:** Rhizosphere soil fungi alpha diversity indices.

Sample	Observed species richness	Shannon diversity index	ACE	Good’s coverage index (%)
**RMS1**	1794a	6.73a	2218.8a	98.87
**RMS3**	1349b	6.18 a	1597.2ab	99.23
**RMS5**	1043b	5.62b	1386.1b	99.43

Values are means ± standard error (n = 3). Means followed by the same letter for a given factor are not significantly different (*P* < 0.05; LSD test)

### Fungal taxa and relative abundance in continuous cropping rhizosphere soil of *C*. *chinensis*

**Fig 2:**
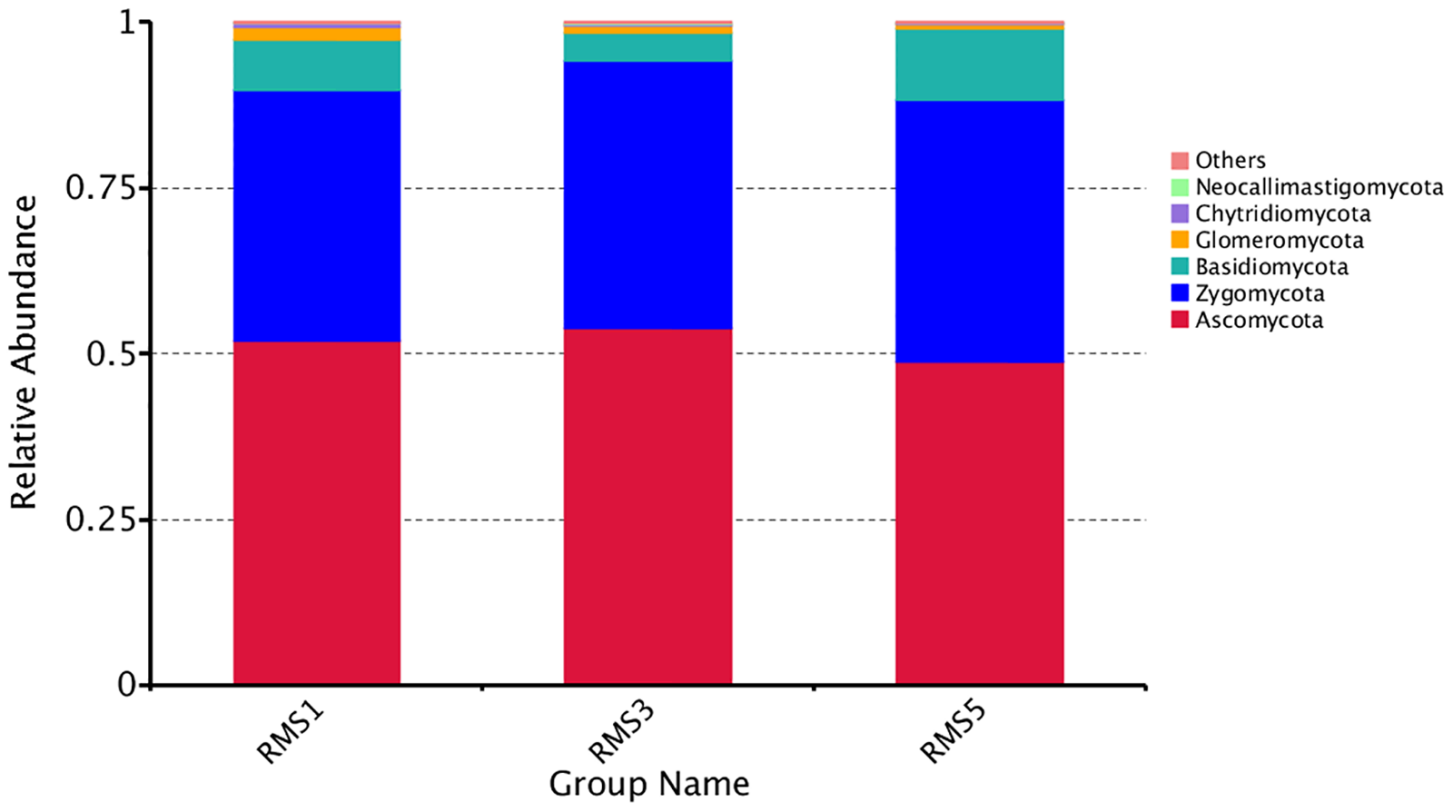
Composition of the different fungal phyla in different continuously cropped soil samples. “Others” represents the sum of the relative abundances of all phyla except the 6 listed.

In the rhizosphere soil samples, all the sequences were classified into six fungal phyla using the Mothur program. The overall fungal composition of the three treatments was similar; Ascomycota, Zygomycota, Basidiomycota, and Glomeromycota were the dominant phyla (Fig 2) and had relative abundances > 0.01. ANOVA showed that the relative abundance of Basidiomycota in RMS3 was significantly lower than in RMS1 and in RMS5 (*P* < 0.05). The relative abundance of Glomeromycota in RMS1 was significantly higher than that in RMS3 and RMS5 (*P* < 0.05). No significant differences in the relative abundances of Ascomycota or Zygomycota were observed among the three samples (*P* > 0.05) (S2 Table).

**Fig 3:**
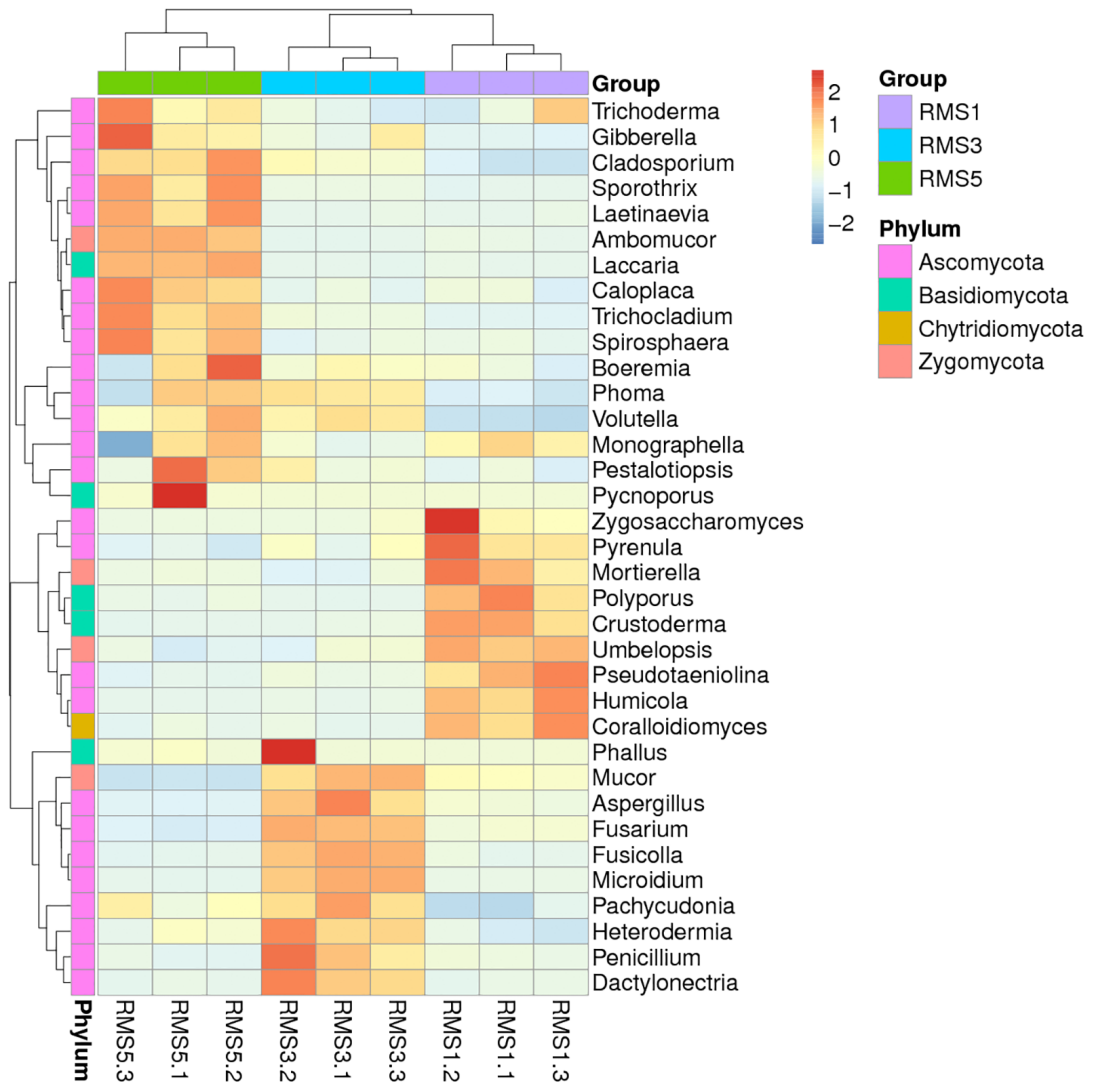
Distribution of the 35 most abundant fungal genera among the three *C*. *chinensis* rhizosphere soil samples. Samples are listed on the Y-axis and fungi species on the X-axis. For the heat map, the left side of the cluster tree is the species cluster tree and the above cluster tree is a sample cluster tree. The mediate heat map represents the Z value of each line, which was calculated as the difference in the average relative abundances of fungi genera from all samples divided by their standard deviations.

For a better and direct illustration of the results, particularly the similarity, variation and relative abundance of fungal composition among the three samples a diagram was produced. Among the fungal samples, the 35 most abundant fungal genera were plotted as a heat map diagram (Fig 3).

As shown in Fig 3, the relative abundances of the genera *Zygosaccharomyces*, *Pseudotaeniolina*, *Hydnum*, *Umbelopsis*, *Humicola*, *Crustoderma*, *Psilocybe*, *Coralloidiomyces*, *Mortierella*, *Polyporus*, *Pyrenula*, and *Monographella* were higher in RMS1 than in RMS3 or RMS5. The proportions of the genera *Sporothrix*, *Trichocladium*, *Caloplaca*, *Spirosphaera*, *Laccaria*, *Ambomucor*, *Pycnoporus*, *Laetinaevia*, *Gibberella*, *Cladosporium*, *Trichoderma*, and *Volutella* were higher in RMS5 than that in RMS1 or RMS3, whereas the proportions of the genera *Fusarium*, *Aspergillus*, *Penicillium*, *Dactylonectria*, *Fusicolla*, *Microidium*, *Heterodermia*, *Phallus*, *Pachycudonia*, *Mucor*, and *Phoma*, were higher in RMS3 than in RMS1 or RMS5. The genera *Phoma*, *Volutella*, *Pachycudonia*, *Heterodermia*, *Cladosporium*, *Gibberella*, *Trichocladium*, and *Sporothrix* had higher proportions in RMS3 and in RMS5 than in RMS1. Overall, the results indicate that continuous cropping of *C*. *chinensis* leads to the increase in the relative abundances of the genera *Phoma*, *Volutella*, *Pachycudonia*, *Heterodermia*, *Cladosporium*, *Phallus*, *Gibberella*, *Trichocladium*, and *Sporothrix* and a decrease in the relative abundances of the genera *Zygosaccharomyces*, *Pseudotaeniolina*, *Hydnum*, *Umbelopsis*, *Humicola*, *Crustoderma*, *Psilocybe*, *Coralloidiomyces*, *Mortierella*, *Polyporus*, *Pyrenula*, and *Monographella* (Fig 3 and S4 Table).

### Sample clustering analysis

The samples were clustered according to their dissimilarity, whereby RMS3 and RMS5 grouped together and were separated from RMS1 (Fig 3), indicating the soil fungal community structure can be affected by continuous cropping of *C*. *chinensis*.

### Correlation between fungal community composition and environmental factors

**Fig 4:**
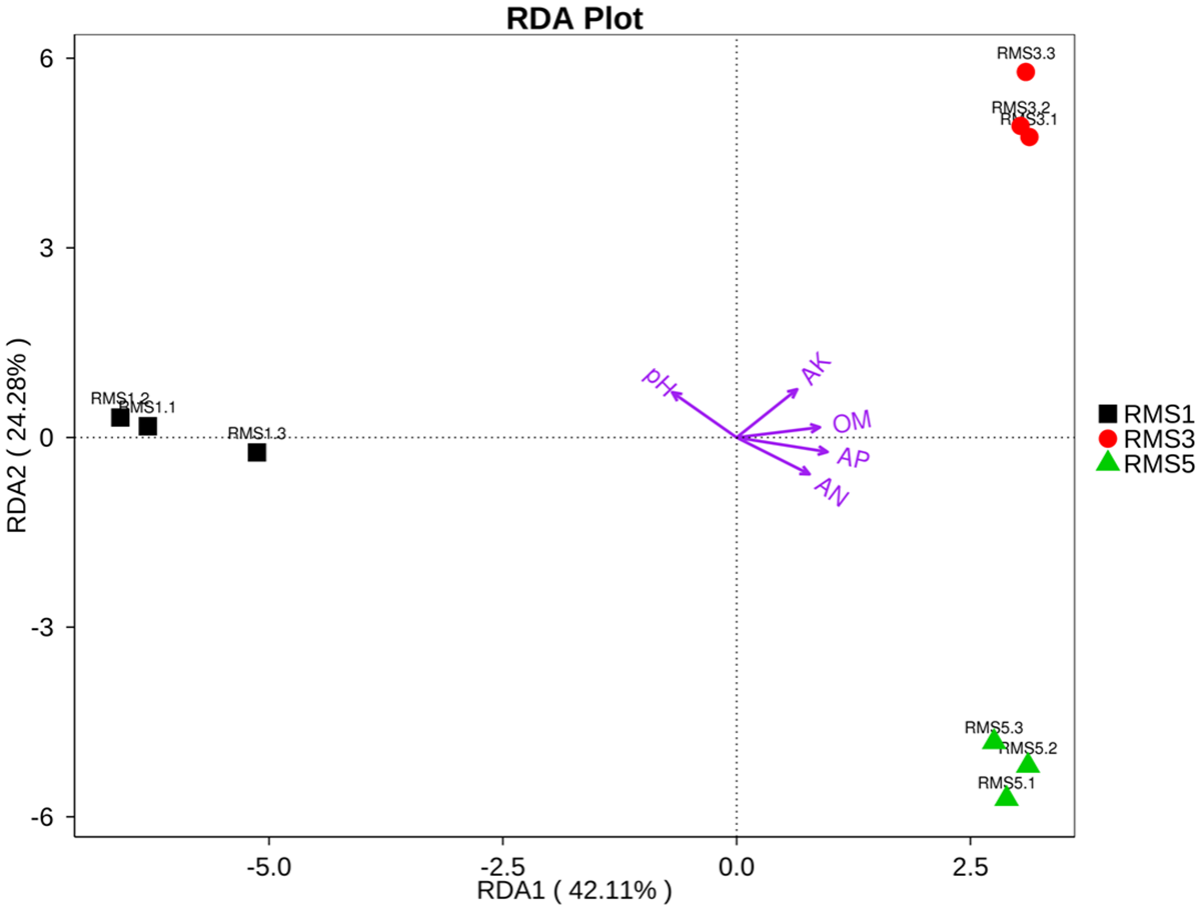
Redundancy analysis of the effects of soil properties and the fungal community on the genus. pH: pH value; OM: soil organic matter; AN: alkaline-hydrolyzable nitrogen; AK: available potassium; AP: available phosphorus.

The results of the detrended correspondence analysis showed that the fungal community composition responded to the soil physicochemical factors following a linear model. This finding indicates that a further study of the fungal communities associated with the soil environment factors is necessary. Thus, a correlation based on the RDA was conducted (S3 Table). In Fig 4, RDA1 separated RMS1 from RMS3 and RMS5, whereas RDA2 separated RMS3 from RMS5. The pH value showed the most significant effects on the fungal community composition (Fig 4).

## Discussion

Continuous cropping problem, also known as continuous cropping obstacle, involving roots, is common to the cultivation of Chinese medicinal herbs [18]. Studies of this problem focused on evaluating the decreasing trend of soil nutrients [42,43]. However, increasing studies have shown that the soil nutrient did not decrease under continuous cropping. In this study, we also observed that available nutrients, such as alkaline-hydrolyzable nitrogen, available phosphorus, and available potassium, all showed an increasing trend with the increase in continuous cropping years. Similar results were also obtained from cropping fields under long-term orchard cropping of black pepper [27], *Pseudostellaria heterophylla* [18] and wild *Rehmannia glutinosa* [44]. Moreover, several researchers believed that the accumulation of allelochemicals under field conditions is a cause of continuous cropping obstacle [45]. However, other researchers opposed this assumption [18]. At present, more studies focus on detecting the roles of soil microbial communities and their relationships with soil environmental factors [27].

The richness and variation of the microbial population plays key role in the sustainable development of soil quality, function and ecosystem [46]. In this study, Illumina HiSeq sequencing of ITS1 showed considerable change of the rhizosphere soil fungal community structure and diversity of *C*. *chinensis* under continuous cropping. The number of OTUs, Shannon diversity index and ACE declined continuously with the increase in the number of continuous cropping years. Zhang [45] also detected that the total microbial biomass and number of fungi decreased significantly with the increase in continuous cropping years by phospholipids fatty acid analysis.

Compared with the traditional 454 pyrosequencing technology, the Illumina HiSeq sequencing technology can detect species diversity efficiently and precisely [47,48]. In the present research, the fungal ITS1 region as analyzed by Illumina HiSeq sequencing provided detailed insights into fungal community patterns in rhizosphere soil of *C*. *chinensis* under continuous cropping. A total of 477,893 effective tags were detected from the three rhizosphere soil samples (S1 Table). The detection of a large number of effective tags indicates that this high-throughput sequencing technique is suitable for analyzing the fungal community composition and their evolution in rhizosphere soils of *C*. *chinensis* under continuous cropping.

Soil microbial communities play essential roles in soil organic matter dynamics and nutrient cycling in agro-ecosystems and have been used as soil quality indicators [25,37]. To some extent, changes in the composition of soil microbial communities (e.g., community types) or in microbial biomass can lead to changes in soil quality [49]. The results of the present study indicated that Ascomycota, Zygomycota, Basidiomycota, and Glomeromycota were the dominant fungal phyla in rhizosphere soil under continuous cropping of *C*. *chinensis* (Fig 2). At the genus level, this research also showed that continuous cropping of *C*. *chinensis* resulted in the increase in the relative abundances of *Phoma*, *Volutella*, *Pachycudonia*, *Heterodermia*, *Cladosporium*, *Gibberella*, *Trichocladium*, and *Sporothrix* and a decrease in the relative abundances of *Zygosaccharomyces*, *Pseudotaeniolina*, *Hydnum*, *Umbelopsis*, *Humicola*, *Crustoderma*, *Psilocybe*, *Coralloidiomyces*, *Mortierella*, *Polyporus*, *Pyrenula*, and *Monographella*.

The clustering analysis and RDA indicated a considerable variation in the fungal community structure across the three samples (Figs 3 and 4). This result consistent with the findings of Bai et al. [50] and Tan et al. [36], which showed that the rhizosphere soil fungal community composition and structure were significantly different based on the number of continuous cropping years.

Meanwhile, investigations of the microbial community in rhizosphere soil could provide new opportunities to explore the potential of antagonistic microorganisms for use in the suppression of plant pathogens [35,51]. *Fusarium* is a pathogen causing root rot in traditional Chinese herbs [18,52]. Previous studies focused on *Trichoderma* because this genus includes species well known for their biological control properties [53–55]. In the present research, the relative abundances of *Fusarium* were 0.22%, 0.60%, and 0.07% in RMS1, RMS3, and RMS5, respectively. Moreover, the proportions of *Trichoderma* were 18.61%, 16.20%, and 22.97% in RMS1, RMS3, and RMS5, respectively (S4 Table). The relative abundances of *Fusarium* and *Trichoderma* indicated lower proportions of root rot in one-year and five-year continuous cropping of *C*. *chinensis* than that in three-year continuous cropping. Changes in *Fusarium* and *Trichoderma* levels in the rhizosphere soil may be associated with root rot in *C*. *chinensis* plants.

Luo et al [56] first reported that *Fusarium solani* was one of the fungal pathogens responsible for root rot in *C*. *chinens*is cultivated in Shizhu County. However, the dominant species in the present study was *Fusarium oxysporu*m, which showed relative abundance percentages of 0.17%, 0.57%, and 0.07% in RMS1, RMS3, and RMS5, respectively. Furthermore, the relative abundances of *F*. *solani* were only 0.04%, 0.02%, and 0.0023% in RMS1, RMS3, and RMS5, respectively (S5 Table). *F*. *oxysporum* is a well-described soil-borne fungus [57] responsible for root rot in many traditional Chinese herbs [18,52]. The high relative abundance of *F*. *oxysporum* in the samples showed that it may be a potential pathogen contributing to the challenges experienced in *C*. *chinensis* continuous cropping system.

Furthermore, knowledge of the soil physicochemical properties and soil enzyme activities in *C*. *chinensis* fields under continuous cropping systems could lead to a better understanding of soil productivity and health. Moreover, detecting the connection between the diversity of soil fungal community and the soil environmental factors could provide direct insight into the mechanisms of continuous cropping obstacle [19,27]. Specific constituents of soil nutrients (e.g., C, N, P and K) and pH may impose physiological constraints on fungal survival and growth, thereby directly altering fungal community composition [58]. Of these constituents, soil pH has been proven to be the most influential factor [59]. In this study, the RDA between the soil physiochemical properties and the fungal community at the genus level showed that the pH of rhizosphere soil had the most significant effect on the fungal community composition. Correlation analysis showed that pH positively correlated with the relative abundance of Ascomycota, Glomeromycota, Chytridiomycota, and Neocallimastigomycota, but negatively correlated with the relative abundance of Basidiomycota and Zygomycota (S1 Fig).

Soil enzymes are involved in the cycling of soil biological elements and the development of soil fertility [24]. Therefore, soil enzyme activity is an important index of soil quality and ecosystem stability [60]. Urease catalyzes the hydrolysis of urea to produce ammonia and carbamate and is an important indicator of soil health. In this study, the activity of urease showed a significant and continuous decline with the increase in continuous cropping years. This result is consistent with the results obtained for the continuous cropping of the pea (*Pisum sativum* L.) [61] and sweet wormwood (*Artemisia annua* L.) [38] and for the monocropping of peanut (*Arachis hypogaea* L.) [62]. Catalase can break down hydrogen peroxide into molecular oxygen and water and thus preventing reactive oxygen species from damaging the cells. In the present study, the catalase activity decreased significantly with the increase in continuous cropping years of *C*. *chinensis* (*P* < 0.05). The decline in catalase activity may cause the increase in the accumulation of toxic substances in the plant roots of *C*. *chinensis* as the number of years of continuous cropping year increases.

## Conclusion

In this paper, Illumina high-through sequencing of ITS1 amplicon showed great change in fungal community composition and diversity of rhizosphere soil under different continuous cropping years of *Coptis chinensis*. pH, urease, and catalase activities of the rhizosphere soil showed a continuous significant decline with the increasing continuous cropping years. The results also indicated that fungal abundance and population diversity decreased continuously with the increase of continuous cropping years. The soil pH had the greatest impact on fungal community composition in rhizosphere soil under continuous cropping of *C*. *chinensis*. Whether *Fusarium oxysporum* is a potential pathogen, which cause the root rot of *C*. *chinensis* will depend on the prospective research.

## Supporting information

S1 Table Effective tags from the three of *C*. *chinensis* rhizosphere soil samples. (DOC)

S2 Table Composition of different fungal phyla in the three of *C*. *chinensis* rhizosphere soil samples. (DOCX)

S3 Table Detrended correspondence analysis (DCA) value. (DOCX)

S4 Table The relative abundance of the 35 most abundant fungal genera among the three of *C*. *chinensis* rhizosphere soil samples. (DOCX)

S5 Table The relative abundance of *Fusarium oxysporum* and *Fusarium solani*. (DOCX)

S1 Fig Correlation between soil physicochemical properties and fungal phylum. (PDF)
