# Supplementary material for: Composition and diversity of rhizosphere fungal community in Coptis chinensis Franch. continuous cropping fields
Source: PLoS One. 2018 Mar 14;13(3):e0193811. doi: 10.1371/journal.pone.0193811 (PMC5851603; doi:10.1371/journal.pone.0193811)
Supplement: S1 Table — (DOC) [file pone.0193811.s001.doc]

**S1 Table.** Effective tags from the three of *C. chinensis* rhizosphere soil samples

| Sample Name | Raw PE | Raw Tags | Clean Tags | Effective Tags |
| --- | --- | --- | --- | --- |
| RMS1.1 | 53,733 | 51,462 | 51,231 | 49,329 |
| RMS1.2 | 51,971 | 48,989 | 48,735 | 46,735 |
| RMS1.3 | 63,766 | 61,930 | 60,040 | 57,979 |
| RMS3.1 | 51,379 | 50,466 | 50,357 | 48,596 |
| RMS3.2 | 55,697 | 54,653 | 54,466 | 52,538 |
| RMS3.3 | 63,463 | 61,527 | 61,310 | 59,119 |
| RMS5.1 | 60,882 | 59,892 | 59,754 | 57,230 |
| RMS5.2 | 60,347 | 59,437 | 59,328 | 57,071 |
| RMS5.3 | 60,160 | 59,196 | 51,370 | 49,305 |
