# Supplementary material for: Composition and diversity of rhizosphere fungal community in Coptis chinensis Franch. continuous cropping fields
Source: PLoS One. 2018 Mar 14;13(3):e0193811. doi: 10.1371/journal.pone.0193811 (PMC5851603; doi:10.1371/journal.pone.0193811)
Supplement: S2 Table — (DOCX) [file pone.0193811.s002.docx]

S2 Table. Composition of different fungal phyla in the three of *C. chinensis* rhizosphere soil samples

| Taxonomy | RMS1.1 | RMS1.2 | RMS1.3 | RMS3.1 | RMS3.2 | RMS3.3 | RMS5.1 | RMS5.2 | RMS5.3 |
| --- | --- | --- | --- | --- | --- | --- | --- | --- | --- |
| Ascomycota | 0.508888 | 0.487209 | 0.563865 | 0.534493 | 0.569279 | 0.512791 | 0.455626 | 0.507399 | 0.500947 |
| Zygomycota | 0.383685 | 0.401123 | 0.346959 | 0.412493 | 0.368661 | 0.424517 | 0.416531 | 0.377594 | 0.390453 |
| Basidiomycota | 0.079589 | 0.081890 | 0.067835 | 0.038666 | 0.047961 | 0.044780 | 0.115209 | 0.102576 | 0.10199 |
| Glomeromycota | 0.019717 | 0.020461 | 0.014483 | 0.010152 | 0.009362 | 0.012227 | 0.010603 | 0.009136 | 0.002527 |
| Chytridiomycota | 0.006497 | 0.006542 | 0.005550 | 0.001624 | 0.001850 | 0.002098 | 0.001421 | 0.002075 | 0.002504 |
| Neocallimastigomycota | 0.000654 | 0.001241 | 0.000384 | 0.001534 | 0.001714 | 0.002030 | 9.02E-05 | 0.00018 | 9.02E-05 |
| Others | 0.000970 | 0.001534 | 0.000925 | 0.001038 | 0.001173 | 0.001557 | 0.000519 | 0.001038 | 0.001489 |
