# Supplementary material for: Composition and diversity of rhizosphere fungal community in Coptis chinensis Franch. continuous cropping fields
Source: PLoS One. 2018 Mar 14;13(3):e0193811. doi: 10.1371/journal.pone.0193811 (PMC5851603; doi:10.1371/journal.pone.0193811)
Supplement: S3 Table — (DOCX) [file pone.0193811.s003.docx]

S3 Table. Detrended correspondence analysis (DCA) value

|  | DCA1 | DCA2 | DCA3 | DCA4 |
| --- | --- | --- | --- | --- |
| Axis length | 1.026525489 | 0.519040905 | 0.50653735 | 0.455336053 |
|  | 1.530457441 | 0.703966094 | 0.452753996 | 0.452682353 |
