# Supplementary material for: Composition and diversity of rhizosphere fungal community in Coptis chinensis Franch. continuous cropping fields
Source: PLoS One. 2018 Mar 14;13(3):e0193811. doi: 10.1371/journal.pone.0193811 (PMC5851603; doi:10.1371/journal.pone.0193811)
Supplement: S4 Table — (DOCX) [file pone.0193811.s004.docx]

S4 Table. The relative abundance of the 35 most abundant fungal genera among the three of *C. chinensis* rhizosphere soil samples

| Taxonomy (Genus) | Relative abundance (%) | | |
| --- | --- | --- | --- |
|  | RMS1 | RMS3 | RMS5 |
| Trichoderma | 18.6105 | 16.1989 | 22.9697 |
| Mucor | 8.0408 | 15.3124 | 1.4287 |
| Trichocladium | 0.1346 | 0.5271 | 2.6184 |
| Umbelopsis | 3.153 | 2.5702 | 2.4905 |
| Zygosaccharomyces | 0.6835 | 0.0707 | 0.015 |
| Crustoderma | 1.1415 | 0.0684 | 0.0293 |
| Aspergillus | 0.203 | 0.7933 | 0.0534 |
| Pycnoporus | 0.0023 | 0.0008 | 0.3166 |
| Phoma | 0.0917 | 0.7061 | 0.5828 |
| Phallus | 0.0038 | 0.2978 | 0.0368 |
| Cladosporium | 0.1466 | 0.3564 | 0.652 |
| Microidium | 0.0752 | 0.7174 | 0.0391 |
| Fusarium | 0.2196 | 0.6031 | 0.0744 |
| Humicola | 0.4407 | 0.0211 | 0.0075 |
| Spirosphaera | 0.0978 | 0.0887 | 0.3557 |
| Pseudotaeniolina | 0.3196 | 0.0384 | 0.0128 |
| Laccaria | 0.012 | 0.0038 | 0.3346 |
| Gibberella | 0.0256 | 0.0925 | 0.2241 |
| Monographella | 0.2602 | 0.1549 | 0.2121 |
| Sporothrix | 0.0128 | 0.0323 | 0.2624 |
| Penicillium | 0.0985 | 0.2534 | 0.0775 |
| Ambomucor | 0.0384 | 0.0263 | 0.3023 |
| Fusicolla | 0.0211 | 0.2557 | 0.0098 |
| Mortierella | 0.191 | 0.0376 | 0.0534 |
| Polyporus | 0.191 | 0.0045 | 0.012 |
| Volutella | 0.0248 | 0.1722 | 0.1737 |
| Pyrenula | 0.1617 | 0.0699 | 0.0353 |
| Dactylonectria | 0.0083 | 0.1654 | 0.006 |
| Caloplaca | 0.0293 | 0.0256 | 0.1466 |
| Pachycudonia | 0.0346 | 0.1361 | 0.0842 |
| Laetinaevia | 0.0045 | 0.0038 | 0.1248 |
| Coralloidiomyces | 0.1173 | 0.0083 | 0.0083 |
| Heterodermia | 0.0105 | 0.1143 | 0.0376 |
| Psilocybe | 0.1226 | 0.0165 | 0.0165 |
| Hydnum | 0.1286 | 0.0218 | 0.0143 |
