# Supplementary material for: Composition and diversity of rhizosphere fungal community in Coptis chinensis Franch. continuous cropping fields
Source: PLoS One. 2018 Mar 14;13(3):e0193811. doi: 10.1371/journal.pone.0193811 (PMC5851603; doi:10.1371/journal.pone.0193811)
Supplement: S5 Table — (DOCX) [file pone.0193811.s005.docx]

S5 Table. The relative abundance of *Fusarium oxysporum* and *Fusarium solani*

| Taxonomy (Species) | Relative abundance (%) | | |
| --- | --- | --- | --- |
|  | RMS1 | RMS3 | RMS5 |
| *Fusarium oxysporum* | 0.1677 | 0.5715 | 0.0707 |
| *Fusarium solani* | 0.0444 | 0.0165 | 0.0023 |
