# Supplementary material for: Composition and diversity of rhizosphere fungal community in Coptis chinensis Franch. continuous cropping fields
Source: PLoS One. 2018 Mar 14;13(3):e0193811. doi: 10.1371/journal.pone.0193811 (PMC5851603; doi:10.1371/journal.pone.0193811)
Supplement: S1 Fig — (PDF) [file pone.0193811.s006.pdf]

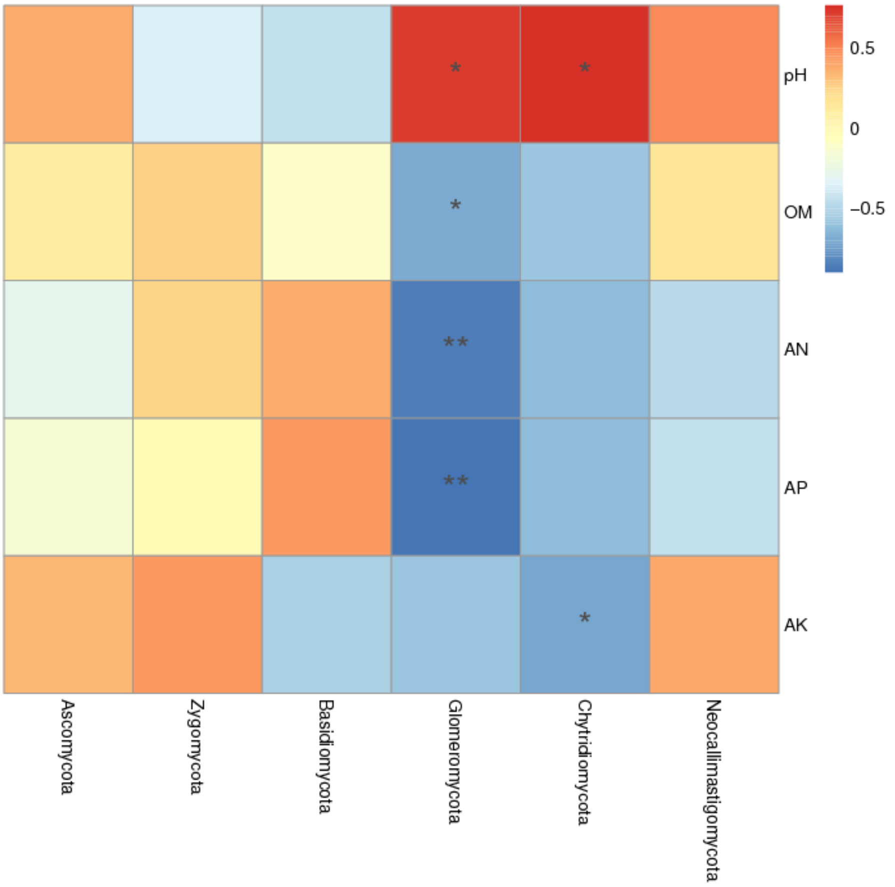

S1 Fig. Correlation between soil physicochemical properties and fungal phylum

pH: pH value; OM: soil organic matter; AN: alkaline-hydrolyzable nitrogen; AK: available potassium; AP: available phosphorus.
